# Factors That Contribute to the Mental Health of Black Youth during COVID-19 Pandemic

**DOI:** 10.3390/healthcare12121174

**Published:** 2024-06-11

**Authors:** Bukola Salami, Aloysius Nwabugo Maduforo, Olivia Aiello, Samah Osman, Oserekpamen Favour Omobhude, Kimberly Price, Jo Henderson, Hayley A. Hamilton, Janet Kemei, Delores V. Mullings

**Affiliations:** 1 Cumming School of Medicine, University of Calgary, Calgary, AB T2N 1N4, Canada; 2 Faculty of Nursing, University of Alberta, Edmonton, AB T6G 1C9, Canada; olivia.aiello@mail.utoronto.ca (O.A.); sosman2@ualberta.ca (S.O.); fomobhude123@gmail.com (O.F.O.); pricekim601@gmail.com (K.P.); 3 Margaret and Wallace McCain Centre for Child, Youth and Family Mental Health, Centre for Addiction and Mental Health, Toronto, ON M6J 1H4, Canada; jo.henderson@camh.ca; 4 Institute for Mental Health Policy Research, Centre for Addictions and Mental Health, Toronto, ON M6J 1H4, Canada; hayley.hamilton@camh.ca; 5 Faculty of Nursing, Grant McEwan University, Edmonton, AB T5J 4S2, Canada; kemeij@macewan.ca; 6 Equity, Diversity, Inclusion and Anti-Racism, Memorial University of Newfoundland, St. John’s, NL A1C 5S7, Canada; dmullings@mun.ca

**Keywords:** Black, youth, COVID-19, mental health, racial disparities, anti-Black racism

## Abstract

Background: The mental health of Black youth during the COVID-19 pandemic is potentially influenced by various systemic factors, including racism, socioeconomic disparities, and access to culturally sensitive mental health support. Understanding these influences is essential for developing effective interventions to mitigate mental health disparities. Methods: Our project used a community-based participatory (CBP) research design with an intersectional theoretical perspective. An advisory committee consisting of fourteen Black youth supported all aspects of our project. The research team consisted of experienced Black researchers who also trained six Black youths as research assistants and co-researchers. The co-researchers conducted individual interviews, contributed to data analysis, and mobilized knowledge. Participants were recruited through the advisory committee members and networks of Black youth co-researchers and sent an email invitation to Black community organizations. Forty-eight Black identified were interviewed between the ages of 16 and 30 in Canada. The data was analyzed thematically. We kept a reflexive note throughout all aspects of the project. Results: Participants reported significant challenges with online schooling, including a lack of support and access to resources. Lockdowns exacerbated stress, particularly for those living in toxic living/home environments. Financial burdens, such as food insecurity and precarious employment, were prevalent and exacerbated mental health challenges. Additionally, experiences of anti-Black racism and police brutality during the pandemic heightened stress and anxiety among participants. Conclusions: The findings underscore the complex interplay of systemic factors in shaping the mental health of Black youth during the COVID-19 pandemic. Addressing these disparities requires targeted interventions that address structural inequities and provide culturally competent support to mitigate the impact on mental well-being.

## 1. Introduction

### 1.1. Introduction

According to World Health Organization [1,2], mental health refers to a person’s emotional, psychological, and social well-being. It encompasses various aspects of life, including how individuals think, feel, and act, as well as how they handle stress, relate to others, and make decisions. Good mental health encompasses more than the lack of mental issues; it includes possessing positive attributes, resilience, and effective coping skills for life’s challenges [1,2]. Factors influencing mental health include biological factors, life experiences, and family history [1,2,3]. Mental health is crucial for overall well-being and affects one’s ability to form relationships, work productively, and make sound decisions. It is a dynamic and multifaceted aspect of human health.

Black youth’s mental health is influenced by racism, socioeconomic factors, and access to support. Studies show racial discrimination correlates with psychological distress [4]. Socioeconomic disparities exacerbate challenges, affecting access to resources and contributing to stress [5]. Family support and community networks mitigate these effects, fostering resilience [6]. However, systemic barriers, like inadequate access to culturally relevant mental health services, persist [7]. Promoting mental health among Black youth necessitates addressing systemic inequities, amplifying their voices, and cultivating inclusive environments [8].

The mental health disparities faced by Black youth during the COVID-19 pandemic have emerged as a critical area of concern, necessitating a nuanced exploration of the multifaceted factors influencing their well-being. Banks [9] illuminates the impact of the pandemic on Black adolescents in the Southeastern United States, revealing insights into their mental health experiences and engagement with mental health services. Despite grappling with both the positive and negative effects of the pandemic, these adolescents underscore the pivotal roles of family and peers in navigating stressors. Persisting challenges include a lack of access to mental health services compounded by financial constraints and domestic disruptions such as family conflicts, relationship problems, financial difficulties, substance abuse issues, domestic violence, parenting challenges, grief and loss, and housing instability [9]. Okoro et al. [10] conducted a comprehensive assessment of the COVID-19 impact on the Black and African American community, shedding light on effects on mental health conditions, access to healthcare, and social well-being. Structural inequities such as poverty, homelessness, and underemployment exacerbate mental health challenges and healthcare disparities within this population, necessitating racially and culturally responsive interventions [10]. Meanwhile, Salami et al. [11] delved into the mental health factors affecting Black youth in Alberta, Canada, uncovering a multitude of stressors, including racial discrimination, the intergenerational gap in families, microaggression and stigma, academic expectations, financial stress, a lack of identity, previous traumatic events, and religion. The study emphasizes the urgency of addressing anti-Black racism and implementing equitable strategies to support the mental well-being of Black youth in Canada [11].

The existing literature points to the significance of family and peer relationships, the impact of systemic inequities, and the role of cultural factors in shaping the mental health experiences of Black youth [9,10,11,12]. However, a comprehensive understanding of how these factors intersect and influence mental health outcomes among Black youth is lacking. Furthermore, the urgent calls for transformative changes in mental health services [9] and the need for culturally responsive interventions [10,12] underscore the imperative to explore practical and context-specific strategies that can effectively address the mental health disparities faced by Black youth.

This study therefore answered the question of how various factors influence the mental health outcomes of Black youth in Canada during the COVID-19 pandemic. This study proposes to fill the existing research gap by investigating the nuanced and intersecting factors that influence the mental health of Black youth during the COVID-19 pandemic. By exploring the experiences of Black youth in different geographical and cultural contexts, the research seeks to contribute valuable insights that can inform targeted interventions, policies, and support systems tailored to the unique needs of this vulnerable population. The overarching problem addressed by this study is the insufficient understanding of the complex dynamics shaping the mental health outcomes of Black youth during the pandemic, hindering the development of effective strategies to mitigate mental health disparities within this demographic group. This research aims to explore the factors influencing the mental health of Black youth during the COVID-19 pandemic in Canada, contributing valuable insights to the discourse on mental health disparities and guiding the development of targeted interventions to support the well-being of Black communities. The research will play a crucial role in developing mental health interventions during a pandemic or health emergency.

### 1.2. Theoretical Framework

This study is grounded in intersectionality theory, which examines the interconnected nature of various forms of social oppression at societal, interpersonal, and individual levels [13,14]. By employing an intersectionality perspective [15], we analyze how factors such as gender, race, class, and other social identities influence the mental health of Black youths. This approach allows us to consider issues of power and our own positionalities in the research process.

Intersectionality theory, first conceptualized by Kimberlé Crenshaw [16], provides a critical framework for understanding how multiple social identities and the corresponding oppressions overlap and interact. This theory is crucial in highlighting the complexity of social inequities and their compounded impact on marginalized groups. In this study, we utilize intersectionality to dissect how intertwined social categories such as gender, race, and class contribute to the unique mental health challenges faced by Black youths. By grounding our research in Critical Race Theory (CRT), we emphasize the pervasive influence of systemic racism and its intersections with other forms of social stratification. CRT posits that racism is not just a series of individual acts but is deeply embedded within legal systems, policies, and social institutions [17]. This theoretical lens is instrumental in uncovering the structural and institutional barriers that exacerbate mental health disparities among Black youths.

Through this research, we aim to extend the discourse on intersectionality within CRT by providing empirical evidence that highlights the necessity of considering multiple intersecting identities when addressing mental health issues. This comprehensive approach ensures that interventions and policies are more inclusive and effective in mitigating the adverse effects of social oppression on Black youths.

## 2. Methodology

Our research utilized a community-based participatory (CBP) approach informed by Critical Race Theory (CRT) and an intersectional framework. By incorporating intersectionality, which is a key concept within CRT, we analyzed how various social factors such as race, gender, and income intersect to shape health outcomes for Black youth. Intersectionality, rooted in CRT, places a central focus on race and power dynamics [15]. We are also cognizant that these experiences are situated along power axes. We go beyond single-issue framing and also seek to capitalize on the agency of Black youth as productive members of society. By incorporating CRT and emphasizing intersectionality, our discourse on racism extends beyond mere acknowledgment to actively promote racial justice. This includes a nuanced understanding of power relations and reflexivity, highlighting the importance of relationality in addressing systemic inequalities.

The CBP research involves researchers working with a specific identified community towards the emancipatory goal of empowering individuals within that community to address systemic barriers and advocate for meaningful social change [18,19]. We followed our earlier approach to CBP research with Black youth, with a focus on community engagement, decolonization, and empowerment [20]. The impetus for this project is our earlier work with Black youths, which identified barriers to accessing mental health services in Alberta, and further research exploring the multifaceted factors influencing the mental health of Black youth in the region, including issues such as racial discrimination, intergenerational tensions, academic pressures, and positive contributors like mental health openness and supportive relationships [11,21]. At the beginning of the project, we set up a 14-member advisory committee of Black youth comprised of intersectional representations (age, gender, sexual orientation, (dis)ability, health, language, and region). Six Black youth were hired as research assistants and co-researchers. These youth collected data, supported data analysis, and contributed to knowledge mobilization (including presentation, guest speaking, and co-authorship on this manuscript). Black youth research assistants met with a postdoctoral fellow on the project every week for effective mentorship. Advisory committee members also met with the team approximately every three months.

We obtained ethics approval from the University of Alberta Research Ethics Board. We recruited participants through advisory committee members on the project, Black youth co-researchers, and advertisements to Black community organizations across Canada. We interviewed Black youths aged 16 to 30 who live in Canada between February 2022 and May 2022. We conducted qualitative interviews with 48 Black youth to understand the factors that contributed to their mental health during the COVID-19 pandemic. Our sample size was based on data saturation. Interviews were conducted online (via Zoom) and were open-ended and semi-structured interviews. Interviews occurred after participants had fully read an informed consent document and had their questions answered. Interviews were semi-structured, lasted approximately 1 h, and were audio-recorded and transcribed verbatim. Participants also completed a demographic form.

Data collection and analysis for interviews occurred iteratively. Audio-recorded data was transcribed verbatim. Data analysis was completed using thematic analysis aided by NVivo 12 qualitative data analysis software. Thematic analysis is a method for identifying, analyzing, and reporting patterns within a dataset [22]. Our thematic analysis encompasses several steps: (1) familiarizing ourselves with data through repeated readings of the transcripts; (2) generating initial codes; (3) searching for themes based on identified codes; (4) reviewing, expanding, and refining identified themes; (5) defining and naming the themes; and (6) writing the final report [22]. The intersectional theory enabled us to consider intersecting and multifactorial influences on the mental health of Black youth. We also kept a reflexive memo to examine our positionalities and our emerging awareness in the field.

## 3. Results

The 48 Black-identifying participants interviewed were between the ages of 16 and 30 years old, with 25 male and 23 female participants, of whom 38 were Christians, 4 were Muslims, and 6 were non-religious or other. Our findings highlight the structural and systemic factors of the COVID-19 pandemic, the main themes being: 1. experience of online schooling; 2. experiencing lockdowns and the sub-theme of the closures of recreational and sports centers; 3. financial burdens, which demonstrated three sub-themes: food insecurity, precarious employment and housing, and lack of funding from the government; and 4. experiencing intersections of anti-Black racism, police brutality, and COVID-19. These factors are unique to the COVID-19 pandemic and have contributed to mental health challenges for Black youth. 

### 3.1. Experience of Online Schooling

The experience of virtual learning and online schooling is a notable factor in the COVID-19 pandemic. Participants describe the challenges of online studies, lack of access to technology and resources to support learning, and lack of support from teachers. The first quote showed the lack of motivation experienced by participants in online educational environments during the pandemic.

“Anyways, I’m in a bio class, and it is impossible to learn online. There’s just so much to it—there’s just so much to it, that not having a face-to-face, like raising my hand or asking the question, it is just a lot harder online. Just watching like prerecorded lectures and finding the motivation, it is yeah, it is definitely been stressful school-wise”.

(Male, 20; born in Kenya)

Online schooling caused high stress levels in participants. They found that teachers were not supportive.

“With COVID considering the online studies we take, I feel some teacher’s kind of just take advantage of it because they say we at home will for sure have nothing to do since it is online to give assignments in class”.

(Female, 18, born in Cameroon)

As most of our participants were students in post-secondary education, they found that the closures of the institutions had an impact on their mental health and their ability to navigate their studies. Their wellness practices were taken away. 

Without support from their teachers and conflicts associated with online learning, participants’ statements demonstrate that they struggled with stress and ongoing mental health challenges. This led to a lack of motivation, lowered grades, and low self-esteem, along with a lack of available coping and low wellness practices to deal with the stress of school to begin with.

### 3.2. Experiencing Lockdowns

Lockdowns and physical distancing from social engagements were highly challenging systemic factors of the COVID-19 pandemic that largely impacted mental health. With schools closed and Black youth receiving less support than usual, as mentioned, participants experienced high stress and a lack of motivation. Participants highlighted how Black youth experienced specific challenges, such as living in toxic households during the lockdowns, which disproportionately impacted their mental health.

“I think having so many lockdowns, not just for black youth, but in general, is really hard on mental health, right? Because you’re at home. You’re socially isolated. Especially for people who—like if we’re thinking about Black youth or the black population in general, certain people who do not live in safe home environments, or live in toxic households, or have parents who are abusive, or—situations like that, which black youth and black individuals would be more likely to be in, being in a lockdown probably could be, like physically unsafe for them, to be in that home environment for such an extended period of time, but also negatively impact their mental health”.

(Female, 23, Born in Jamaica)

This statement demonstrates concerns about the detrimental effects of multiple lockdowns on mental health, emphasizing the challenges faced by Black youth in unsafe home environments. Prolonged lockdowns are seen as potentially physically unsafe and damaging to the mental well-being of Black youth in these situations. This could be linked to poverty or family breakdown [23]. 

### 3.3. Recreational and Sports Closures

Due to the lockdowns and lack of support in school, Black youth were without the resources that were necessary to cope with COVID-19. This is especially the case for the closure of recreational centers like gyms and basketball courts. The closures impacted Black youth by preventing them from being able to access sports and after school activities that would have greatly helped their mental health during the isolation and hopelessness of the pandemic. These spaces being closed contributed to the lack of sports and physical activity, which participants state took a hit on the mental health of Black youth. Especially for Black youth, participants mentioned the importance of the basketball community, and these closures set up consequences for their mental health.

“I do know that in the basketball community and where there is a lot of young black youth that was harshly affected, in the sense that sport is a vector of change through these certain underserved communities and basketball, it was basically all they had or the main thing that was driving them to wake up in the morning and go play hard and yeah, I’ll do school also. At least they have basketball, and that was definitely one sort of aspect of life for these young black youth that I know took a hit and probably took a toll on their mental health as well.”

(Male, 24, Born in Rwanda)

Thus, the closure of recreational facilities impacted a central source of resilience for Black youths, sports. Participants discussed the discrimination along lines of race in the development and enforcement of policies. For instance, in some provinces, bars were allowed to remain open while recreational facilities were forced to close. Participants stated there were nuances of anti-Black racism that they interpreted seeing the closures of basketball courts, which were predominantly utilized by Black youth.

“You know what, honestly, one spot in which I thought it really would have an effect on black youth was by my house, where there’s the basketball court. And, you know, it is a predominantly black neighborhood; I go there. And when that was like closed down during the early stages of the pandemic, it was like—it was one of the more strictly enforced places like in the entire neighborhood. Like the playground was not nearly as hardly enforced as the basketball court, I felt like that was almost targeted to some extent.”

(Male, 22, born in the Netherlands)

Participants’ statements surrounding the over-surveillance of spaces where Black youth may frequent, such as basketball courts, demonstrate another barrier experienced by Black youth that non-Black youth and communities did not. In the research of Evans and Francis (2020), over-surveillance by the police was found to be another intersection of oppression experienced by Black youth that was exacerbated by the COVID-19 pandemic. 

### 3.4. Experiencing Financial Burdens

Participants spoke about financial factors that impacted their mental health. They spoke of three sub-themes: food insecurity, job and housing precarity, and the lack of sustainable funding support from the government, which specifically impacted Black youth. 

#### 3.4.1. Food Insecurity

Participants spoke about being food insecure and having to navigate the challenges of also paying rent. They had minimal resources to access during the pandemic, so they had to be creative and find their own support. This was especially the case for youths who have multiple intersecting vulnerabilities, including race and disability:

“So yeah, I noticed throughout the pandemic I liked—I was not in a good place in terms of my credit but like just stuff happening. Well, financial instability. The food prices are going up. That’s been horrible. On disability, I make 1685, like after rent bills, and all that jazz. I just feel like—I feel kind of frustrated because I feel that, as a person with a disability, I should not be punished. I should still be able to have a good quality of life. That was a big problem for the second year of the pandemic because I would not have money for the last week or the last two weeks of the month. I’d usually just like—it was the summer, so I would usually just go to random places, and sometimes they’d be like giving out free pizza on [unintelligible 00:22:47]. I do not know if you go to church and like to eat a snack or something.”

(Female, 24, born in Canada)

Issues of poverty and food insecurity are mentioned in the literature on how Black youth in particular were impacted by the pandemic [23,24]. 

#### 3.4.2. Precarious Employment and Housing

Within the same vein, participants talked about housing challenges; not being able to pay rent, losing housing, or finding themselves homeless because they were not able to pay rent and were not supported by the government. They center specifically on Black youth and Black people experiencing poverty and dealing with ongoing financial burdens that were exacerbated during the pandemic.

“But I feel like the pandemic affected several youths, and some lost their job, some lost their means of livelihood, and some had to—some even lost their homes, because of not being able to keep up with rent.”

(Male, Born in Canada)

These statements identify the secular barriers and financial factors of COVID-19 that impacted Black and racialized people during the pandemic more than non-Black people, including poverty and financial challenges. Black youth were implicated in these challenges as their families were impacted (Wallace, 2020 [24]), and their mental health suffered. 

#### 3.4.3. Barriers to Government Financial Support

The government of Canada provided financial support to individuals and families during the pandemic, such as the Canada Emergency Response Benefit (CERB), offering financial assistance to employed and self-employed Canadians directly impacted by COVID-19. Recipients received $2000 per 4-week interval (equivalent to $500 weekly) from 15 March to 26 September 2020 [25]. Thus, participants focused on the limited access to government funding, or CERB, during the pandemic, stating how this limited and residual form of funding caused a lot of worry and mental health problems.

“If you do not have access to CERB, then you have a lot of worries and, like, a lot of mental health-related problems, you know, stemming from, you know, worrying about your financial situations and that kind of precarity around that time. I can also see how—and then even with just getting vaccinated and not—and if you’re undocumented, for example, and being worried about, you know, are you going to be deported or detained.”

(Male, 23, Born in Jamaica)

This comment summarizes the way the lack of adequate financial support from the government, precarious employment and housing, and food insecurity were not experienced in isolation but rather were connected to the COVID-19 pandemic, and particularly for Black people, they were also connected to anti-Black racism and police brutality, which were impacting Black people collectively across the board during the pandemic, which further exacerbated mental health challenges. 

### 3.5. Experiencing Anti-Black Racism, Police Brutality, and COVID-19

Systemic issues of anti-Black racism and police brutality were brought to the forefront during the height of the COVID-19 pandemic when George Floyd was killed in May 2020, which spurred the largest Black Lives Matter movement in North America. The intersection of these experiences impacted Black youths’ mental health.

“But yeah, in general, like for Black youth—and that was also like the situation with the Black Lives Matter movement and the George Floyd incident that happened in 2020 that has created a lot of stress for Black youth. You know, just like—like I remember the day after the incident happened, I would be scared, just to walk on the street and see a police car. [Laughs] I’m always so scared of, you know, “Oh, are they going to, you know, try to arrest me for walking or something?” I would just—it just caused me so much stress. I would stay at home as much as possible because I did not want any trouble or anything. So, yeah”.

(Female, Born in Canada)

“And also, like, during the pandemic, there was the whole Black Lives Matter movement, the protests, and everything. And that was a very big moment as well. I know those protests that, like, were held here as well in Edmonton, and I know a lot of people were just fed up. I think the pandemic just accelerated all those emotions as well, because it is like, How many things are we going to deal with? Like, at least with the pandemic, it is—a lot of it is beyond our control, it is physical, and it is to do with our health. But with, you know, racism and with all the issues that police brutality and black people are facing, that’s obviously social, and it is due to other humans.”

(Female, 19, born in the United Kingdom)

The looming mental health stress of the pandemic, alongside multiple incidents of anti-Black racism and police brutality, most notably the killing of George Floyd, demonstrated how Black youth and Black people were experiencing “multiple pandemics” (Bernard, 2020 [26]). This was a strong factor that impacted the mental health of Black youth and, collectively, Black people. 

## 4. Discussion

The first theme that emerged in the research was the experience of online learning. Participants experienced numerous challenges with distance learning, including, higher stress levels, low motivation, difficulty focusing, and poor mental and emotional health. Verlenden et al. [27] conducted a probability-based survey of parents of children aged 5–12 in the US to understand if changes in modes of instruction during the pandemic have presented psychosocial stressors. They found that virtual instruction might present more mental health and emotional health risks than in-person instruction [27]. The findings of our study support the body of literature suggesting that online instruction poses more risks than in-person learning related to a child’s mental and emotional health and wellbeing. The findings of our study also suggest that mental health challenges faced by students because of online learning were further exacerbated as they felt that their wellness practices were taken away due to the containment strategies put in place by the government to slow the spread of the virus. Schools are essential to supporting children not only by way of education, but also through opportunities to participate in activities that support cognitive, psychosocial, and physical health and wellbeing. Therefore, the disruption of school-based services and extracurricular activities contributed to the worsening of mental health outcomes among Black youth and adolescents. Additionally, the experience of stress coupled with the lack of educator support that the youth reported had several negative psychological impacts. Educators and school counselors are important emotional supports for youth and adolescents, as they are often the first to note warning signs of mental health challenges. As professionals working directly with youth, educators have an ethical responsibility to recognize warning signs and intervene when a mental health crisis arises. However, the transition to online learning reduced their ability to provide emotional support, observe crisis warning signs, and interfere with youth at risk during a time when students needed them more than ever before. Finally, our findings suggest that students experienced higher levels of stress related to the challenges of online learning during the pandemic. Early-life toxic stress can negatively affect the physical and mental health of children, especially Black children, who are more likely to experience racial discrimination, poverty, unstable housing, or residential segregation and less likely to have access to social and economic supports [28]. Children who experience early-life toxic stress are at risk of long-term adverse health outcomes that may not present themselves until adulthood. Adverse health outcomes include negative coping mechanisms, poor stress management, unhealthy lifestyle choices, mental and emotional health issues, and chronic illnesses such as cardiovascular disease, cancer, asthma, and depression [29]. 

The challenges of online learning and the COVID-19 pandemic are particularly detrimental to Black students, as they are already at a higher risk of dropping out of secondary school education and are less likely to apply to post-secondary education [30,31,32]. Consequently, the mental health struggles and issues related to online learning and the pandemic may exacerbate an already tenuous academic path for Black students. Further research is needed to understand the long-term impacts of the pandemic and online instruction across the lifetimes of Black children and adolescents. 

The second theme that emerged in the research was the theme of experiencing lockdowns and the sub-theme of the closures of recreational and sports centers. Black youth expressed that they did not have the resources that they needed to cope with the pandemic, particularly with the closures of recreational centers like gyms and basketball courts. Recreational physical activity plays an important role in the management of mental health issues, especially depression and anxiety [33]. Zulyniak et al. [34] examine the associations between recreational and non-recreational physical activity and mental health outcomes among Canadian youth. They found that recreational physical activity was associated with positive mental health for youth; however, non-recreational physical activity showed generally small and insignificant associations with mental health outcomes. Such findings emphasize how detrimental recreational facility closure was to Black youth. The youth in our study particularly highlighted the importance of basketball and the basketball community to their mental health. Ogden and Hilt [35] describe how sports serve as an area for social and racial resistance. In thinking with Early [36] our findings show that basketball is a medium through which Black communities express their rebellion and resistance to social and economic oppression. Further, Boyd [37] explains how basketball “create[s] a space of resistance and free expression that announces a relative notion of empowerment, while at the same time acknowledging the racial and class hierarchies that still dominate sports and society as a whole” (p. 133). The inability to play sports then was particularly harmful to the mental health of Black youth. While recreational facility closures impacted all youth, Black youth felt this harm in greater ways as they experienced over-surveillance of spaces where Black youth frequented. As Dryden [38] explains, the attempt to slow the spread of the virus brought together policing and public health. Dryden draws on the destruction of Africville, Nova Scotia, to demonstrate why Black folks are nefarious of public health measures. In the 1960’s, one of the explanations for the destruction of Africville, Nova Scotia, was the public health claim that the community was at a health risk as there was no sewage system. Instead of providing the necessary services, the community was forcibly removed. Evidently, then, the feeling of being targeted as a Black person in the name of public health follows a long history of public health serving as an extension of policing [38,39]. 

The third theme that emerged in our findings was experiencing financial burdens, which were related to issues of food insecurity, precarious employment and housing, and a lack of funding from the government. The COVID-19 pandemic has limned the ways in which health disparities cannot be extricated from socio-economic precarity. Specifically, subjugated populations have been disproportionately affected by the pandemic due to state austerity, ecological insecurity, the hierarchical identity-based composition of homelessness, and the overall unequal allocation of health resources. In 2016, according to the market basket measure of low income, 27% of Black children younger than age 15 in Canada were living in poverty. This proportion was half as high among other children (14%) [40]. Our findings suggest that Black youths’ mental health suffered because of the experience of food insecurity during the pandemic. Dhunna and Tarasuk [41] found that the prevalence of food insecurity is 3.5 times higher in Black households compared to White households across Canada, even after adjusting for factors like immigration status, education level, and homeownership. They concluded that in Canada, the overruling determinant of vulnerability to household food insecurity is whether one is racialized as Black [41]. Dabone et al. [42] assessed the food insecurity conditions of African, Caribbean, and Black populations globally during the COVID-19 pandemic. They found that the COVID-19 pandemic doubly burdened African, Caribbean, and Black populations, both with the disease and food insecurity within Canada and globally. In fact, according to Food Secure Canada [43], it is expected that food insecurity will double from the existing 4.4 million, with disproportionate impacts on Black and other racialized groups. As such, future public policy on food insecurity in Canada post pandemic must consider the role of systemic and institutionalized racism [43]. Furthermore, from February to April 2020, about 5.5 million Canadians employed were affected by the COVID-19 containment strategies that mandated the closure of all non-essential businesses [44]. This included a drop in employment of 3.0 million jobs [44]. As an attempt to reduce the impact of the economic fallout, Canada implemented the Canada Emergency Response Benefit (CERB), which provided financial support to employed and self-employed Canadians who were directly affected by COVID-19 [45]. This financial support, however, was lower than the usual income of many individuals and many households that received it, meaning many households saw a significant reduction in their income [46]. Participants in our study shared that limited access to government funding or CERB during the pandemic caused mental health challenges for them. Canada Food Banks saw an increase in food bank use (20% to 28%) across the country [47]. As such, financial support implemented during the peak of the COVID-19 pandemic was insufficient to prevent increases in poverty and food insecurity. This has been particularly harmful to the mental health of Black youth, who are already disproportionately affected by poverty and food insecurity.

Youth in our study talked about housing challenges, including not being able to pay rent, losing housing, or finding themselves homeless because they were not able to pay rent. Housing is a social determinant of health, and COVID-19 has made it apparent that housing and health are intrinsically interlinked. Nearly five million people in Canada currently live in poverty [48] and prior to the emergence of COVID-19, Canada was facing a homelessness and housing crisis [49]. However, the socio-spatial patterning of the virus indicates that such a crisis is not a mere reflection of an irresponsible, failed liberal subjectivity but rather a direct consequence of an institutional failure to accommodate and preserve lives. Such institutional negligence functions through the social ordering of populations through hegemonic modalities of gender, race, class, sexuality, and ability, making some bodies more vulnerable to precarious housing situations than others. When COVID-19 was officially declared an international pandemic, public health professionals urged Canadians to stay at home and practice “social distancing” to flatten the curve. Access to shelter proves imperative in any attempt to follow such public health advice, emphasizing the importance of housing as a social determinant of health. However, race-based economic inequalities force individuals into tighter dwellings, creating an increased risk of transmission [50,51]. Additionally, overpolicing of racialized communities has also placed Black men and women in the closed quarters of detention centers, which similarly increases the risk of exposure and transmission [52]. In such conditions, people are forced to sleep inches away from each other, creating an environment conducive to the spread of disease and making the isolation of those infected impossible. Our findings show us that housing is health care and has rendered visible the fragilities of the public sector with respect to low-income Black folks.

The final theme that emerged in our findings was experiencing the “multiple pandemics” of anti-Black racism, police brutality, and COVID-19 [26]. In 2020, following the murder of George Floyd by Officer Derek Chauvin, the largest racial justice protests in the United States since the Civil Rights Movement erupted [53]. BLM protests went far beyond American borders; global action was spurred. Black youth in our study discussed how incidents of anti-Black racism and police brutality, most notably the killing of George Floyd, impacted their mental health negatively. Additionally, our findings showed that Black youth live in constant fear of the police. Williams [54] reported that Black youth constantly fearing for their lives, feel hopeless and alienated from society at large, given the lack of support from social institutions such as law enforcement.

The feelings of alienation constantly felt by Black youth are of concern as a sense of belonging is crucial to child development [55]. Since the murder of George Floyd, anxiety and stress among Black populations have increased at higher rates than any other racial group. Suicide rates for Black youth were already rising at startling rates. To such a great degree that Black youth under 13 are two times more likely to die by suicide than their white counterparts [56]. Interventions are needed to meet the post-pandemic mental health needs of Black youth, who are constantly experiencing an epidemic of racism. Such interventions must be guided by accessibility and cultural competency. 

## 5. Limitations

Although this study has provided important insights into the mental health of Black youth, it is important to note that the sample was not representative of the entire population of Black youth in Canada. Specifically, the study had a low representation of Black youth who are involved in the justice system or who are out of school and out of work. The generalizability of our findings to this population is limited.

It is important to recognize that the primary objective of this study was not to achieve generalizability, but rather to facilitate the transferability of our findings. Through the inclusion of verbatim quotes and detailed context in the methodology, we have taken steps to enhance the transferability of our study, with the hope that our findings will inform future research, policies, and regulations aimed at supporting the mental health of Black youth in Canada.

## 6. Implications for Policy and Practice

Our findings underscore several critical areas where current policy and practice can be informed to better meet the needs of Black youth in Canada and globally. The experiences highlighted by our participants reveal the compounded effects of financial burdens, food insecurity, precarious employment and housing, a lack of sustainable government support, and systemic racism during the COVID-19 pandemic. Addressing these intersecting issues requires a comprehensive and multifaceted approach.

Firstly, economic support programs should provide stable financial assistance for Black youth, particularly during and after crises like the COVID-19 pandemic. Policies must ensure access to unemployment benefits, housing subsidies, and food assistance programs to alleviate financial stressors disproportionately affecting Black youth. Specific initiatives targeting food security, stable employment, and accessible housing are paramount to mitigating economic challenges and enhancing mental health outcomes.

Secondly, it is crucial to develop and implement mental health services that are culturally sensitive and accessible to Black youth. Training mental health professionals in cultural competence and addressing systemic barriers that prevent Black youth from seeking help are essential steps. Creating environments where Black youth feel understood and supported will make mental health services more effective in addressing their specific needs.

Thirdly, educational policies need to focus on enhancing the virtual learning experience for Black youth. This includes addressing issues of technology access, providing robust teacher support, and instilling effective coping mechanisms for the challenges posed by online schooling. Initiatives that reduce barriers to higher education and create job opportunities, such as scholarships, internships, and mentorship programs, will help mitigate some of the economic challenges faced by Black youth.

Fourthly, community-based support systems must be strengthened to provide a safety net for Black youth. Funding for community organizations that offer mental health services, mentorship programs, and other resources tailored to the needs of Black communities is vital. These organizations play a critical role in providing support and fostering resilience among Black youth, especially during lockdowns and periods of social isolation.

Additionally, anti-racism and mental health awareness campaigns can play a pivotal role in addressing the interconnected issues of racial disparities, police brutality, and mental health challenges. The COVID-19 pandemic has highlighted the urgent need for these campaigns to combat systemic racism and its detrimental impact on mental health.

Finally, advocating for policy reforms that address systemic racism and its impact on health and wellbeing is crucial. Reforms in healthcare, education, and economic systems are needed to ensure they are equitable and just. This includes addressing the root causes of systemic inequities and implementing policies that promote social justice and equality.

Globally, these findings underscore the importance of international collaboration and knowledge sharing to address the unique challenges faced by marginalized communities during health emergencies. By applying the principles of Critical Race Theory (CRT) and intersectionality, policymakers and practitioners can develop more effective strategies to support Black youth and other marginalized groups worldwide. This holistic approach, combining educational, community-based, financial, awareness, and policy-focused interventions, will foster the mental well-being of Black youth during the unique challenges posed by the COVID-19 pandemic and beyond.

## 7. Conclusions

In conclusion, this study sheds light on the intricate interplay of systemic factors impacting the mental health of Black youth during the COVID-19 pandemic. From the challenges of online schooling and the mental health ramifications of lockdowns to financial burdens and the intersectionality of anti-Black racism, police brutality, and the pandemic, the findings underscore the multifaceted nature of the struggles faced by Black youth. The implications drawn from these insights suggest a comprehensive approach encompassing educational interventions, community support programs, financial assistance initiatives, anti-racism and mental health awareness campaigns, and policy reforms. By addressing these key areas, there is potential to mitigate the mental health disparities experienced by Black youth and foster resilience in the face of adversity. As we move forward, it is imperative to recognize and act upon these insights to create a more equitable and supportive environment for Black youth navigating the challenges of pandemics and difficult situations.

## Data Availability

Individuals interested in accessing the data can contact the first author at oluwabukola.salami@ucalgary.ca.
